# Editorial: Individual differences: from neurobiological bases to new insight on approach and avoidance behavior

**DOI:** 10.3389/fnsys.2015.00125

**Published:** 2015-09-15

**Authors:** Daniela Laricchiuta

**Affiliations:** ^1^IRCCS Fondazione Santa LuciaRome, Italy; ^2^Department of Dynamic and Clinical Psychology, Faculty of Medicine and Psychology, University “Sapienza” of RomeRome, Italy

**Keywords:** affective and emotional neuroscience, dopaminergic and endocannabinoid systems, fear system, motivational disorders, personality traits, reinforcement sensitivity theory, resilience, rewarding and aversive stimuli

Many different labels have been proposed over the years to cover the definition of approach and avoidance. Initially, an Approach-Avoidance distinction was conceptualized in terms of valence-based processes, rather than over behavior. In 1960s, an Approach-Withdrawal distinction was introduced arguing that in all organisms the motivation is grounded in overt behavioral actions toward or away from stimuli. Subsequently, it was presumed that action and emotional tendencies are grounded in specific brain systems. Only recently, it was preferred the Approach-Avoidance distinction that expands the previous Approach-Withdrawal distinction in terms of energization of the behavior by (motivation), or direction of the action toward (behavior), positive stimuli in the case of the approach, and in parallel, energization of the behavior by, or direction of the action away from, negative stimuli in the case of the avoidance (Laricchiuta and Petrosini, 2014).

The approach and avoidance behaviors appear to be the primary reactions to novel, rewarding, or dangerous stimuli on which all successive responses are based in order to gain successful adaptation. Thus, the positive or negative valence of the stimulus is considered the core of Approach-Avoidance distinction. Further, the hedonic principle to approach pleasure and avoid pain is frequently presumed to be the fundamental principle upon which motivation is built (Cornwell et al., 2014). In this framework, the approach system is considered a motivational system that activates reward-seeking behavior associated with impulsivity/exploration, whereas the avoidance system is considered an attentional system that promotes appetitive response inhibition or active overt withdrawal.

The approach and avoidance behaviors are biologically based and constitutionally ingrained, since all organisms are “pre-programmed” to approach or avoid particular classes of stimuli. Approach and avoidance behaviors are anchored to the brain networks implicated in action and reaction to salient stimuli and controlling cognitive and attentional functions, reward sensitivity and emotional expression. These networks involve cerebral nodes interconnected as prefrontal cortex, amygdala, hypothalamus, striatum and cerebellum. By acting on them the neurotrasmitter systems increase the intensity of appetitive or defensive motivation. In fact, individual differences in approach and avoidance behaviors might be modulated by normal variance at the level of functioning of different neurotransmitter systems, such as dopaminergic, serotoninergic, noradrenergic and endocannabinoid systems as well as many peptides such as corticotropin releasing hormone. Experimental findings collected over the years show how the genetic background may play a critical role in modulating aminergic and GABAergic neurotransmission in prefrontal-accumbal-amygdaloid system in response to different rewarding or aversive experiences. Further, important results highlight the modulatory role for genetic variability of the dopaminergic system in individual differences in action-valence interaction (Richter et al., 2014).

Physiologically, human temperaments of approach and avoidance are viewed as instigators of propensity. They produce immediate cognitive, affective, and behavioral inclinations in response to stimuli and orient individuals across domains and situations in a consistent fashion. Although the action undoubtedly emerges directly from the temperamental proclivities, ultimate behavioral outcomes are often function of the integration among goal pursuit, self-regulation, and temperament traits. Also the motivational salience plays an important role in shaping behavior. Individuals regulate their emotions in a wide variety of ways. The aberrations in the elaboration of aversive or rewarding stimuli as well as defective coping strategies characterize many psychopathological disorders, as attention-deficit/hyperactivity disorders, depression and substance abuse on one hand, or anxiety and post-traumatic stress disorder on the other hand. Thus, individual differences in approach and avoidance may represent predictors of vulnerability (or resilience) to neuropsychiatric diseases.

The present Research Topic deals with the hot issue of individual differences in emotional and motivational processing, attempting to clarify “what,” “how,” and “why” of human and animal approach and avoidance behaviors, emphasizing the link between neuronal pattern and behavioral expression (McNaughton and Corr, 2014). The Topic includes experimental and clinical researches on the individual differences focusing behavioral characterization, structural and neurochemical profiles, synaptic connections, and receptor expression of approach and avoidance (Andolina et al., 2015). The translational models included in the present Research Topic consider the neurobiological mechanisms that give rise to outliers in approach and avoidance behaviors (Galatzer-Levy et al., 2014). Using the central tendency that assumes population homogeneity potentially overlooks the individual differences that explain responses to positive or negative stimuli. Crucial findings indicate that the heterogeneous approach and avoidance responses may be informative for understanding both resilience and impaired coping strategy.

Further, great importance has been given to the researches facing the clarification of diseases associated with inappropriate responses to aversive or rewarding situations. An interesting contribution to the Research Topic has been given from a literature revision on Parkinson's disease to understand whether neurobiological (dopaminergic dysfunction) and neuropsychological (executive function alteration) modifications due to Parkinson's disease are associated to changes in approach-avoidance related personality features (Costa and Caltagirone, 2015). Parkinson's disease patients may show approach-avoidance imbalance as documented by lower novelty-seeking and higher harm-avoidance temperamental traits.

It has been also addressed the issue of whether some forms of emotional regulation are healthier than others by focusing on two commonly used strategies: cognitive reappraisal (changing the way one thinks about potentially emotion-eliciting events) and expressive suppression (changing the way one behaviorally responds to emotion-eliciting events) (Cutuli, 2014). Findings on individual differences have been reviewed showing that using cognitive reappraisal to regulate emotions is associated with healthier patterns of affect, social functioning, and well-being in comparison to using expressive suppression. Once more, brain structural basis and functional activation linked to the habitual use of cognitive reappraisal and expressive suppression are discussed in detail.

Given the growing need for standardized paradigms (Markett et al., 2014) and self-report inventories measuring individual differences in approach and avoidance, a new questionnaire measuring the revised constructs of behavioral inhibition and activation systems and fight-flight-freezing system has evidenced that a functional genetic polymorphism on the arginine vasopressin receptor 1a gene is associated with individual differences in the behavioral inhibition dimensions (Reuter et al., 2015).

Considered as a whole, the present Research Topic calls attention on individual differences related to approach and avoidance behaviors as resilience or risk factors to disease and inefficient coping strategies, in response to environmental challenges.

## Conflict of interest statement

The author declares that the research was conducted in the absence of any commercial or financial relationships that could be construed as a potential conflict of interest.
